# The m6A reader protein YTHDC2 is a potential biomarker and associated with immune infiltration in head and neck squamous cell carcinoma

**DOI:** 10.7717/peerj.10385

**Published:** 2020-11-26

**Authors:** Yang Li, Ji-Na Zheng, En-Hao Wang, Chan-Juan Gong, Keng-Fu Lan, XiaoJun Ding

**Affiliations:** 1 Department of Stomatology, Zhongshan Hospital, Fudan University, Shanghai, China; 2 State Key Laboratory of Molecular Engineering of Polymers, Fudan University, Shanghai, China; 3 Department of Dermatology, Zhongshan Hospital, Fudan University, Shanghai, China; 4 Department of Otolaryngology, Union Hospital of Tongji Medical College, Huazhong University of Science and Technology, Wuhan, China

**Keywords:** N6-methyladenosine (m6A), Head and neck squamous cell carcinoma (HNSCC), Weighted gene co-expression network analysis, Survival analysis, YTHDC2, Tumor-infiltrating

## Abstract

**Background:**

Increasing evidence has shown that N6-methyladenosine (m6A) RNA methylation regulators have important biological functions in human cancers. However, there are few studies on the value of m6A reader protein YTHDC2 in the diagnosis and tumor-infiltrating of head and neck squamous cell carcinoma (HNSCC). Therefore, it is important to understand the potential clinical value of YTHDC2 in the prognosis and immune infiltration of HNSCC.

**Methods:**

In this study, gene expression profiles and the corresponding clinical information of 270 HNSCC patients were downloaded from the Gene Expression Omnibus (GEO) database. The gene co-expression network was established to verify whether YTHDC2 was related to the prognosis of HNSCC and verified again in the public database. The correlations between YTHDC2 and immune infiltration was investigated via Tumor Immune Estimation Resource (TIMER) and Gene Expression Profiling Interactive Analysis (GEPIA).

**Results:**

The results showed that YTHDC2 appeared in the blue module related to survival time and survival state and had a close correlation with the prognosis and immune infiltration level of HNSCC in public database. Patients with low expression of YTHDC2 had poor overall survival (OS) and recurrence-free survival (RFS) than those with high expression. In addition, the expression of YTHDC2 was positively correlated with the level of CD4+ T cell subpopulations infiltration in HNSCC.

**Conclusions:**

Through this study, we found that YTHDC2 is a tumor suppressor gene with high expression in normal tissues and low expression in tumor tissues. In addition, YTHDC2 is correlated with the immune infiltrating levels of B cells, CD8+ T cells, CD4+ T cells, neutrophils, and dendritic cells in HNSCC, which may become a potential marker for prognosis and immune infiltration of HNSCC.

## Introduction

Head and neck squamous cell carcinoma (HNSCC) is one of the most common malignant tumors in the world (Ferlay et al., 2010). HNSCC mainly include neck tumors, nasopharyngeal tumors, oral and maxillofacial tumors (Leemans, Snijders & Brakenhoff, 2018). At present, studies have found that tobacco and alcohol are important factors in inducing HNSCC (Jethwa & Khariwala, 2017; Kawakita & Matsuo, 2017). The incidence of HNSCC is also relatively high in the world, which not only directly affects patients’ breathing, eating and language functions, but also causes rapid growth of neck lymph node metastasis with the help of abundant blood supply and lymphatic refluence (De Sousa et al., 2015). The main clinical treatment methods for HNSCC include surgery, radiotherapy and chemotherapy (Cramer et al., 2019).

N6-methyladenosine (m^6^A) methylation is a methylation modification on RNA molecules, which was first discovered in 1974 (Dubin & Taylor, 1975). The m^6^A methylation is a reversible dynamic RNA modification that may affect biological regulation, similar to DNA methylation and protein phosphorylation. Adenosine methylation in mRNA plays a regulatory role by changing mRNA function and affecting gene expression (Meyer & Jaffrey, 2014). The m^6^A modification is mainly related to three types of proteases. The first is m^6^A methyltransferase, whose coding genes are called “Writers”, including methyltransferase like 3 (METTL3), METTL14, METTL16, wilm’s tumor 1-associating protein (WATP). They form a complex that causes the m^6^A methylated group to be written into the RNA (Cui et al., 2017). The second type of protein is m^6^A demethylase, which encodes genes called “Erasers”. These proteins can remove the m^6^A methylated group from RNA, thereby affecting tumor biological processes (Huang et al., 2015). The last group of proteins that bind to the m^6^A methylation site to play specific biological functions, which encodes genes called “Readers”. They can recognize mRNA containing m^6^A and regulate the expression of downstream genes accordingly (Meyer & Jaffrey, 2017). Although RNA m^6^A modifications are shaped in a dynamic and reversible manner by the involvement of methyltransferases and demethylases, the proteins that preferentially identify m^6^A-modified can bind to the methylated RNA and give it specific functions. The YT521-B homology (YTH) family with RNA-binding domains were the first “readers” to bind directly to the m^6^A sites of RNA. Some scholars have found that YTH domain can selectively bind m6A sites in RNA to give YTH domain proteins specific functions. Proteins modified by m^6^A and containing the YTH domain can be identified as: YTH domain-containing 1-2 (YTHDC1-2) and YTH N6-methyladenosine RNA binding protein 1-3 (YTHDF1-3). The YTH family protein YTHDF1-3 and its nuclear members YTHDC1 and YTHDC2 can bind directly to RNA containing m^6^A (Wang et al., 2014).

YTHDC2 can improve the efficiency of the translation and reduce the abundance of mRNA through its helicase and can improve the efficiency of HIF-1*α* mRNA translation (Tanabe et al., 2016), and can adjust the happening of the sperm (Hsu et al., 2017). Recent studies have shown that low expression of YTHDC2 was associated with poor prognosis and correlated with activation of apoptosis and ubiquitin-mediated proteolysis in HNSCC (Zhou et al., 2020). Besides, Zhao & Cui (2019) also found that two m6A methylation “readers”—YTHDC2 and heterogeneous nuclear ribonucleoprotein C (HNRNPC) could construct a prognostic signature and predict the survival time in HNSCC patients.

This study investigated whether YTHDC2 was associated with the diagnosis and prognosis of HNSCC. We comprehensively analyzed the expression of YTHDC2 in HNSCC patients and its relationship with prognosis in the Gene Expression Omnibus (GEO) database and multiple public databases. In addition, we also investigated the association between the expression level of YTHDC2 and immune cell infiltration. This study found that YTHDC2 could act as an independent gene as a HNSCC prognostic marker, and provided a potential relationship between YTHDC2 and the interaction of tumor immune infiltration of HNSCC.

## Materials & Methods

### Validation of prognostic genes by weighted gene co-expression network analysis (WGCNA)

In order to validate YTHDC2 whether associated with the prognosis in HNSCC patients, the datasets of GSE65858 were downloaded from the GEO database. This database contained the gene expression of 270 HNSCC patients, as well as a series of corresponding clinical information (overall survival time, survival status, progression-free survival, age, smoking, tumor type, stage, TNM stage). We analyzed the top 25% of genes with the largest variance and used the “WGCNA” package of R 3.6.1 software (Zhao et al., 2010). Firstly, the scale-free network was constructed by the soft threshold *β* of the appropriate adjacency matrix. With the expression matrix and the estimated optimal *β* values, the co-expression matrix can be built directly. The similarity between genes was calculated according to the adjacency, and the system cluster tree among genes was obtained. According to the standard of hybrid dynamic shear tree, the minimum number of genes in each gene module was set as 30. After determining the gene modules, the characteristic vector value of each module was calculated in turn, and then cluster analysis was carried out to merge the modules close to each other into the new module (height = 0.25). Then, the correlation between the modules and traits was analyzed, and the module containing YTHDC2 were selected for further analysis. In addition, we obtained the gene significance (GS) and module membership (MM). Finally, we found that YTHDC2 was associated with prognosis, which was verified again in the public database.

### Bio-informational analysis of YTHDC2 expression from public database

#### Oncolnc database

We used the survival data of HNSCC patients with different expression levels of YTHDC2 contained in Oncolnc database to establish kaplan-Meier survival curve, so as to study the influence of YTHDC2 expression in HNSCC on prognosis (Gao et al., 2018).

#### Kaplan–Meier plotter database

Kaplan–Meier plotter is a powerful public database that can assess the impact of thousands of genes on the survival for cancers. Sources for the database include GEO, the European Genome-phenome Archive (EGA), and The Cancer Genome Atlas (TCGA) (Lánczky et al., 2016). Kaplan–Meier plotter was used to analyze the correlation between the expression of YTHDC2 and survival time in HNSCC. The 95% confidence interval of the hazard ratio (HR) and *P* values were calculated.

### TIMER database analysis

TIMER database is a public online database, which can analyze the prognosis of cancer information as well as the different tumor immunity infiltration data (Li et al., 2017). We used TIMER database to analyze the expression of YTHDC2 in different types of cancer. In addition, we also analyzed the correlation between immune infiltration abundance of the CD8+ T cells, CD4+ T cells, B cells, neutrophils, macrophages, and dendritic cells with the expression of YTHDC2. In addition, we also make the correlation analysis between YTHDC2 and related genes and markers referenced in prior studies of multiple immune cells in TIMER (Siemers et al., 2017; Danaher et al., 2017; Mousset et al., 2019). Besides, we used TIMER2.0 version to analyzed the correlation between the immune infiltration and the expression of YTHDC2 (Li et al., 2020c). The outcome module of Timer 2.0 can explore the clinical correlation of tumor immune subsets and flexibly select covariables, which may be clinical factors or gene expression, to construct a corrected multivariate Cox proportional hazard models. By inputting immune cell types and gene YTHDC2, TIMER 2.0 will perform Cox regression analysis to create an independent Cox model. In addition, the immune infiltration estimations for users-provided expression profiles by multiple algorithms.

### Gene correlation analysis in GEPIA

Gene expression Profling Interactive Analysis (GEPIA) can be used to analyze the RNA expression data of different cancers and normal tissues from the TCGA database and the Genotype-Tissue Expression (GTEx) (Tang et al., 2017). Therefore, we used GEPIA to perform survival analysis of HNSCC and *P* < 0.05 was statistically significant.

### Gene alteration of YTHDC2 in HNSCC from cBioPortal

We obtained YTHDC2 genetic change information in HNSCC by using cBioPortal (Gao et al., 2013). We draw the OncoPrint schematic diagram to reflect the various types of changes (missense mutation, truncating mutation, amplification, deep deletion, up-regulation and down-regulation of mRNA) of YTHDC2 in 279 HNSCC patients. Next, we constructed the kaplan-Meier survival curve to evaluate the impact of YTHDC2 changes on HNSCC patients’ survival. The histogram showed the alteration frequency of YTHDC2 in HNSCC from different sources and tissues.

### Immunohistochemistry (IHC)

We collected paraffin-embedded tissue blocks confirmed as HNSCC in our hospital for IHC analysis, and a total of 10 pairs of normal tissues and HNSCC tissues were stained. Firstly, paraffin sections were dewavered, and the antigen retrieval was performed, and endogenous peroxidase activity was blocked with 0.3% hydrogen peroxide for 25 min at 37° C. Following antigen retrieval, the tissue sections were incubated with primary antibody YTHDC2 (1: 500; EPR21820–EPR21849, AB220160; Abcam, Inc., Cambridge, MA, USA) at 4° C overnight. The next day, after washing with phosphate buffer saline (PBS) for the third time, then secondary antibody was added to incubate 50 min at room temperature. Then, we used diaminobenzidine (DAB) for staining, and hematoxylin was used to re-dye the nucleus.

## Results

### Co-expression analysis by WGCNA

**Figure 1:**
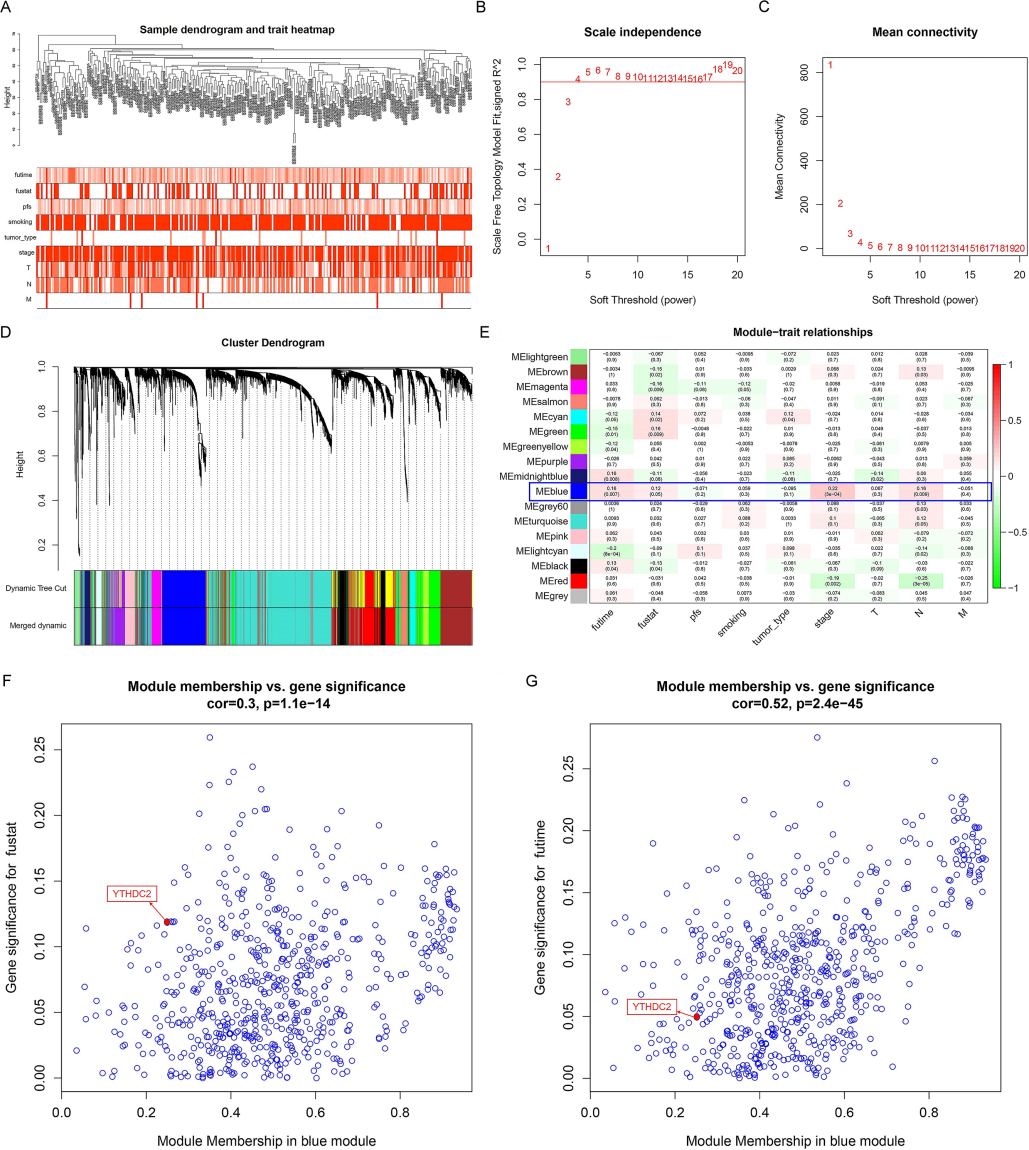
Construction of weighted gene co-expression network construction and identification of the hub module. (A) Clustering dendogram of samples and the clinical traits of overall survival time, survival status, progression-free survival (pfs), age, smoking, tumor type, stage, TNM stage are displayed at the bottom. (B–C) The scale independence and the mean connectivity of the weighted gene co-expression network analysis (WGCNA) analysis of the head and neck squamous cell carcinoma (HNSCC). Testing the scale free topology when *β* = 4. (D) Clustering dendrograms and modules identified by WGCNA. (E) Correlation between module eigengenes and clinical traits. The blue module containing YTHDC2 is selected. (F–G) The scatter diagram of gene significance (GS) vs module membership (MM) in the blue module with survival time and survival state of HNSCC. Gene YTHDC2 has been highlighted in red dots, the cutoff of blue module membership = 0.26, the cutoff of gene significance for survival state = 0.12, and the cutoff of gene significance for survival time = 0.05.

The WGCNA analysis can be used to explore the relationship between gene networks and related phenotypes by establishing co-expressed gene modules. Firstly, the data of 270 HNSCC patients were downloaded from the database GSE65858 (Fig. 1A) and the power of *β* = 4 was chose to construct a co-expression matrix (Figs. 1B and 1C). Finally, we obtained 17 gene co-expression modules. All the modules are represented by different colors, and the gray module showed the genes that cannot be categorized into any module (Fig. 1D). Figure 1E showed the blue module was closely related to the survival time (*P* = 0.007), survival status (*P* = 0.05), stage (P = 3e−04) and lymph node metastasis (*P* = 0.009) of HNSCC patients after the module combined with clinical features. Finally, we drew the scatter diagram of GS vs MM in the blue module. It can be seen here that YTHDC2 was not only highly correlated with its corresponding module, but also highly correlated with its corresponding traits (survival time, survival state) (Figs 1F and 1G).

### The mRNA expression levels of YTHDC2 in different cancer types

**Figure 2:**
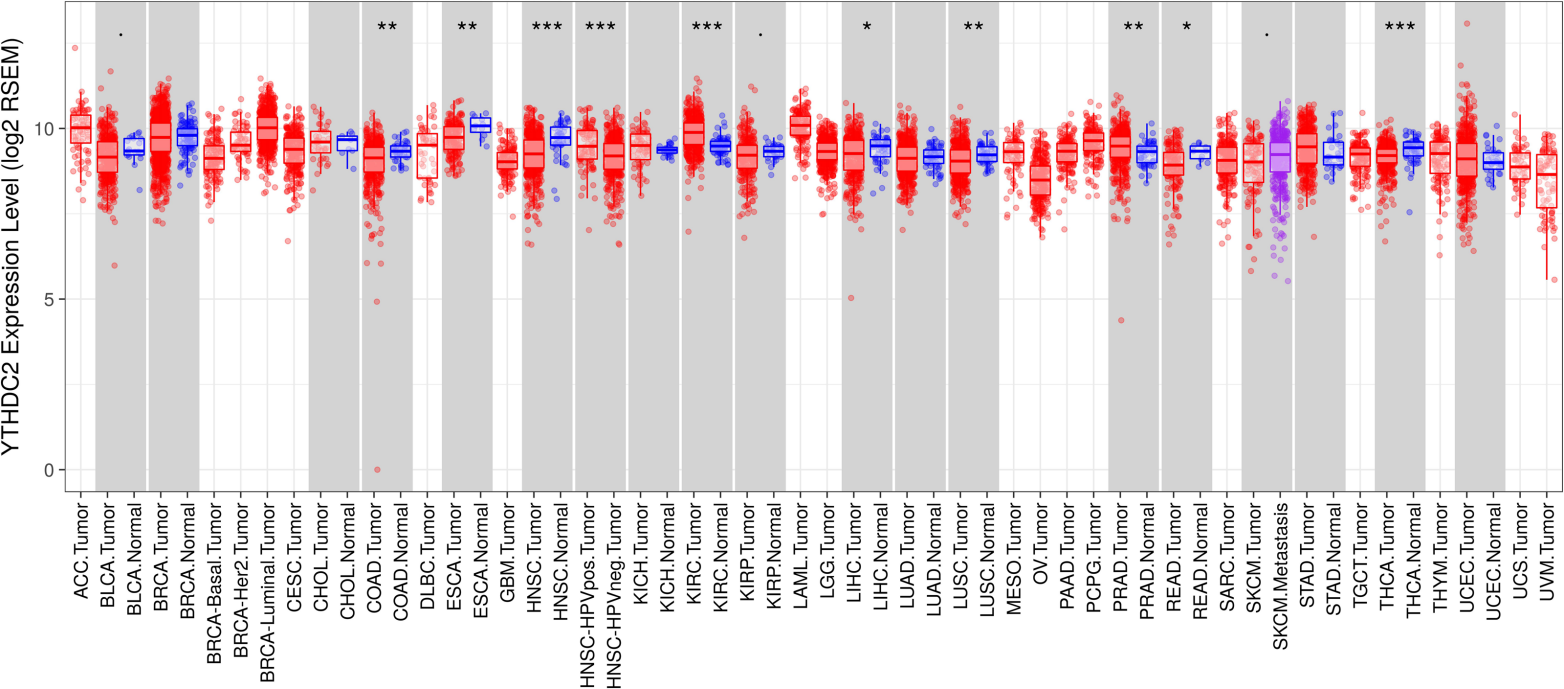
YTHDC2 expression levels in different types of human cancers. (^∗^*P* < 0.05, ^∗∗^*P* < 0.01, ^∗∗∗^*P* < 0.001).

To further evaluate the expression of YTHDC2 in human cancers, we used the RNA-seq data from multiple malignant tumors in TCGA database to detect their expression. Differential expression of YTHDC2 in all TCGA tumors and adjacent normal tissues was shown in Fig. 2. Compared with adjacent normal tissues, the expression of YTHDC2 in HNSCC was significantly decreased (*P* < 0.001).

### Immunohistochemical analysis

**Figure 3:**
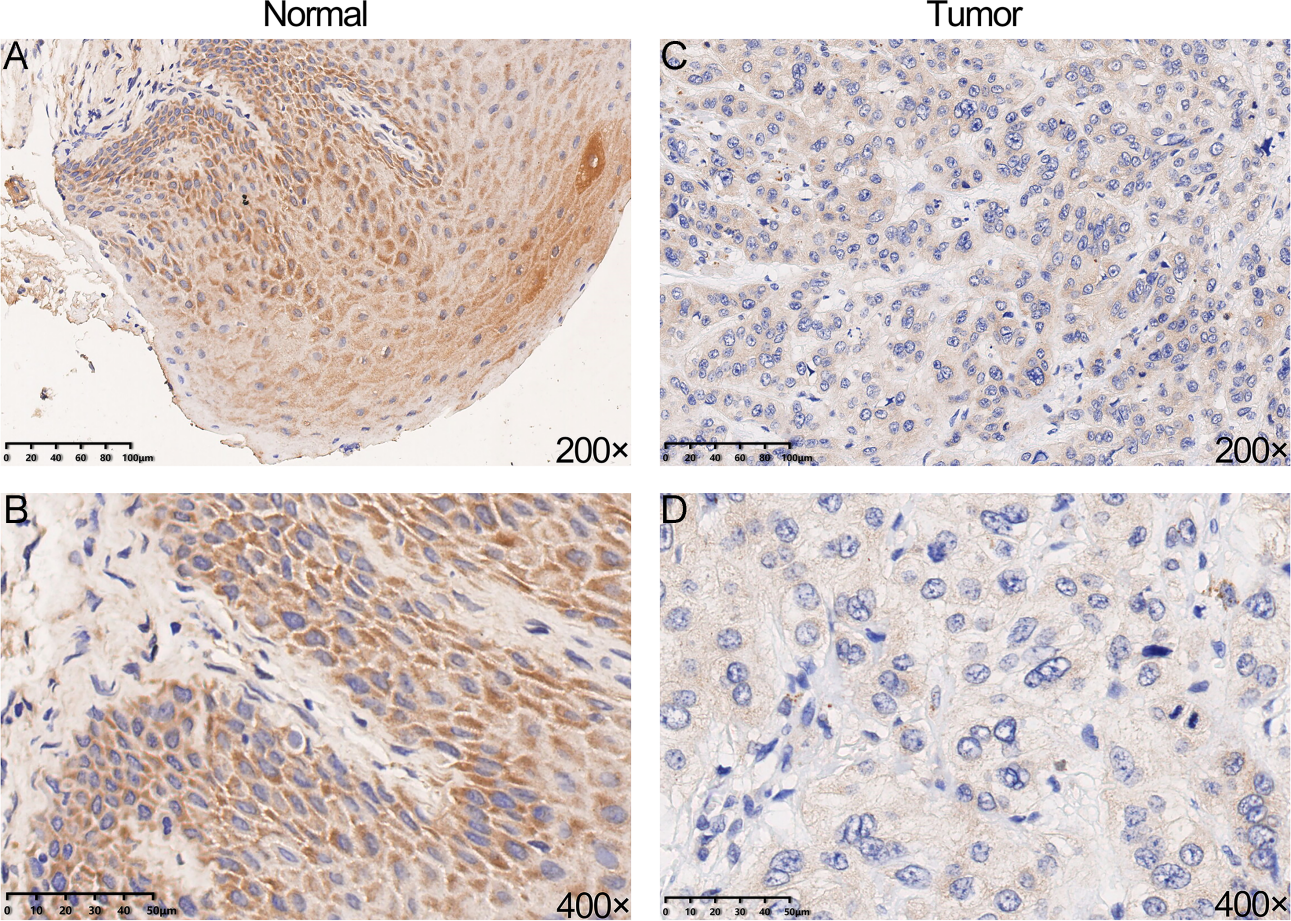
The protein expression of YTHDC2 in head and neck squamous cell carcinoma (HNSCC) and normal oral tissues. (A–B) Normal oral tissues. (C–D) HNSCC tissues.

We performed IHC analysis to confirm whether YTHDC2 was relatively low expressed in tumor tissues, and the result showed the protein expression of YTHDC2 in HNSCC tissue were down-regulated compared with normal tissues, which was consistent with the previous database (Figs. 3A–3D). Based on the above results, we found that YTHDC2 were expressed more highly in normal tissues than in HNSCC.

### The prognostic significance of YTHDC2 expression in HNSCC

**Figure 4:**
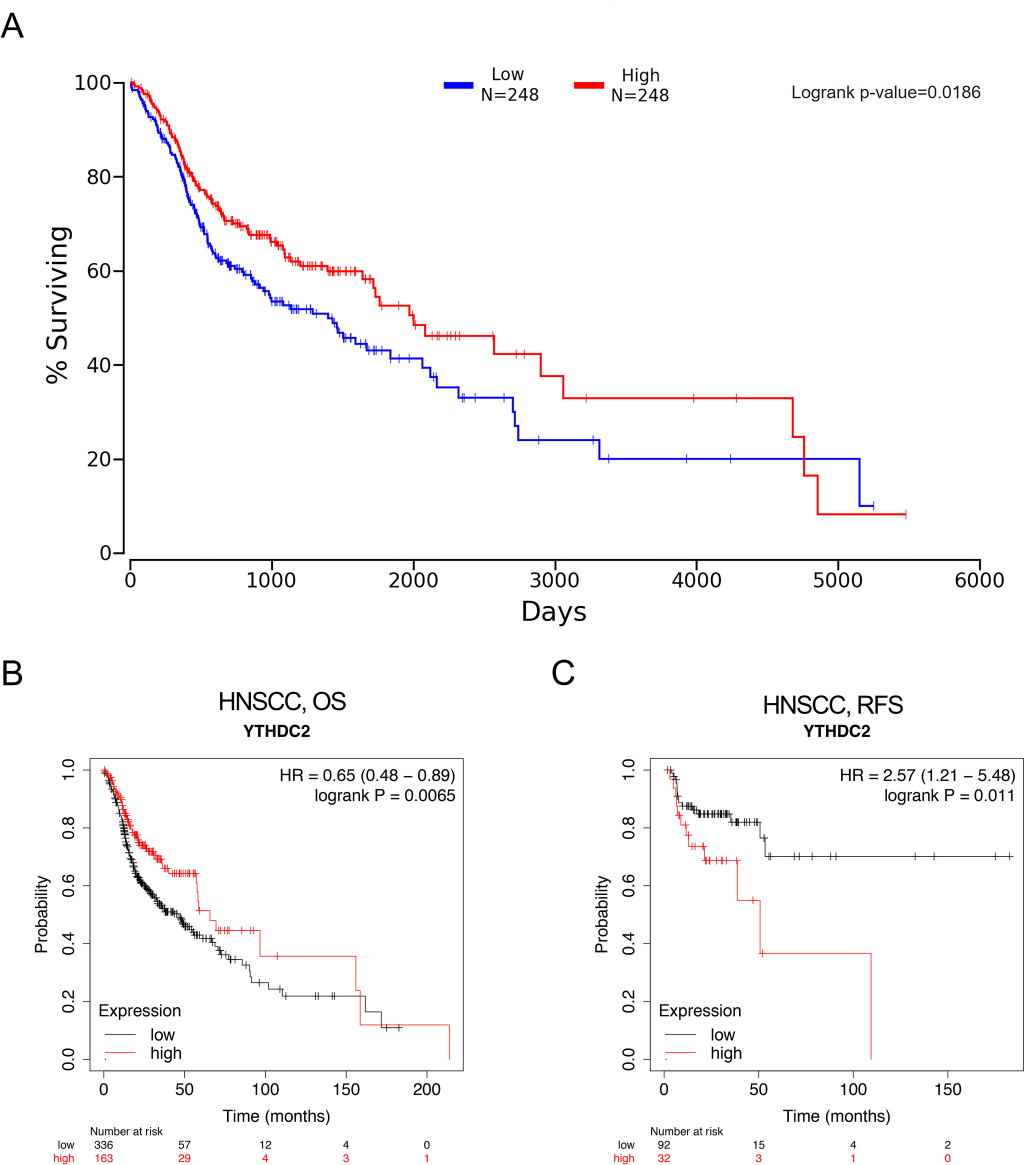
The prognostic significance of YTHDC2 expression in head and neck squamous cell carcinoma (HNSCC) from public database. (A) Kaplan–Meier survival analysis for YTHDC2 expression in head and neck squamous cell carcinoma (HNSCC) patients from Oncolnc database. (B, C) Overall survival (OS) and recurrence-free survival (RFS) survival curves of HNSCC from Kaplan–Meier plotter (*n* = 499, *n* = 124).

Figure 4A showed that HNSCC patients with higher YTHDC2 expression had a better prognosis than patients with lower YTHDC2 expression (*P* = 0.0186) from Oncolnc database. For further verification, we evaluated the prognostic value of YTHDC2 in HNSCC using Kaplan–Meier plotter database, and we found that low expression of YTHDC2 was also associated with poor overall survival (OS) and recurrence-free survival (RFS) (OS HR = 0.65, 95% CI [0.48–0.89], *P* = 0.0065; RFS HR = 2.57, 95% CI [1.21–5.48], *P* = 0.011) (Figs. 4B and 4C). The two databases show consistent results.

### Low expression of YTHDC2 impacts the OS of HNSCC in patients with different clinicopathological factors

We used the Kaplan-Meier plotter database to study the relationship between the expression of YTHDC2 and different clinical characteristics of HNSCC patients. The results showed that low expression of YTHDC2 was associated with poor OS in male patients, grade 1 and grade 2 as well as low mutation burden (*P* < 0.05). In addition, low expression of YTHDC2 was correlated with worse OS in different cellular content (Basophils, B-cells, CD4+ memory cells, CD8+ T-cells, Eosinophils, Macrophages, Mesenchymal stem cells, Natural killer T-cells, Regulatory T-cells, Type 1 T-helper cells, Type 2 T-helper cells) of HNSCC patients (Table 1).

### YTHDC2 correlated with immune infiltration level in HNSCC

**Figure 5:**
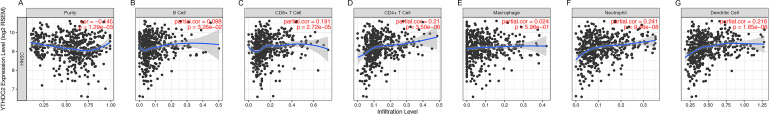
Correlation of YTHDC2 expression with immune infiltration level in head and neck squamous cell carcinoma (HNSCC). (A) Tumor purity (B) B cell (C) CD8+ T cells (D) CD4+ T cells (E) Macrophages (F) Neutrophils (G) Dendritic cells.

Previously, we observed the blue module was closely related to lymph node metastasis (*P* = 0.009) of HNSCC patients (Fig. 1D). Thus, we investigated whether the expression of YTHDC2 was related to the immune infiltration level in HNSCC. We evaluated the correlation between the expression of YTHDC2 with immune infiltration levels in HNSCC from the TIMER. The results showed that the expression of YTHDC2 was significantly correlated with tumor purity (*r* =  − 0.145, *P* = 1.29e−03), B cell (*r* = 0.098, *P* = 3.26e−02), CD8+ T cells (*r* = 0.191, *P* = 2.72e−05), CD4+ T cells (*r* = 0.21, *P* = 3.50e−06), neutrophils (*r* = 0.241, *P* = 9.46e−08) and dendritic cells (*r* = 0.216, *P* = 1.65e−06) in HNSCC (Figs. 5A–5G). In addition, YTHDC2 expression has no significant correlations with the infiltrating levels of Macrophage in HNSCC. These findings strongly suggested that YTHDC2 expression played an important role in the immune infiltration of HNSCC, especially for CD4+ T cells and dendritic cells.

### Correlation analysis between YTHDC2 expression and immune marker sets

**Figure 6:**
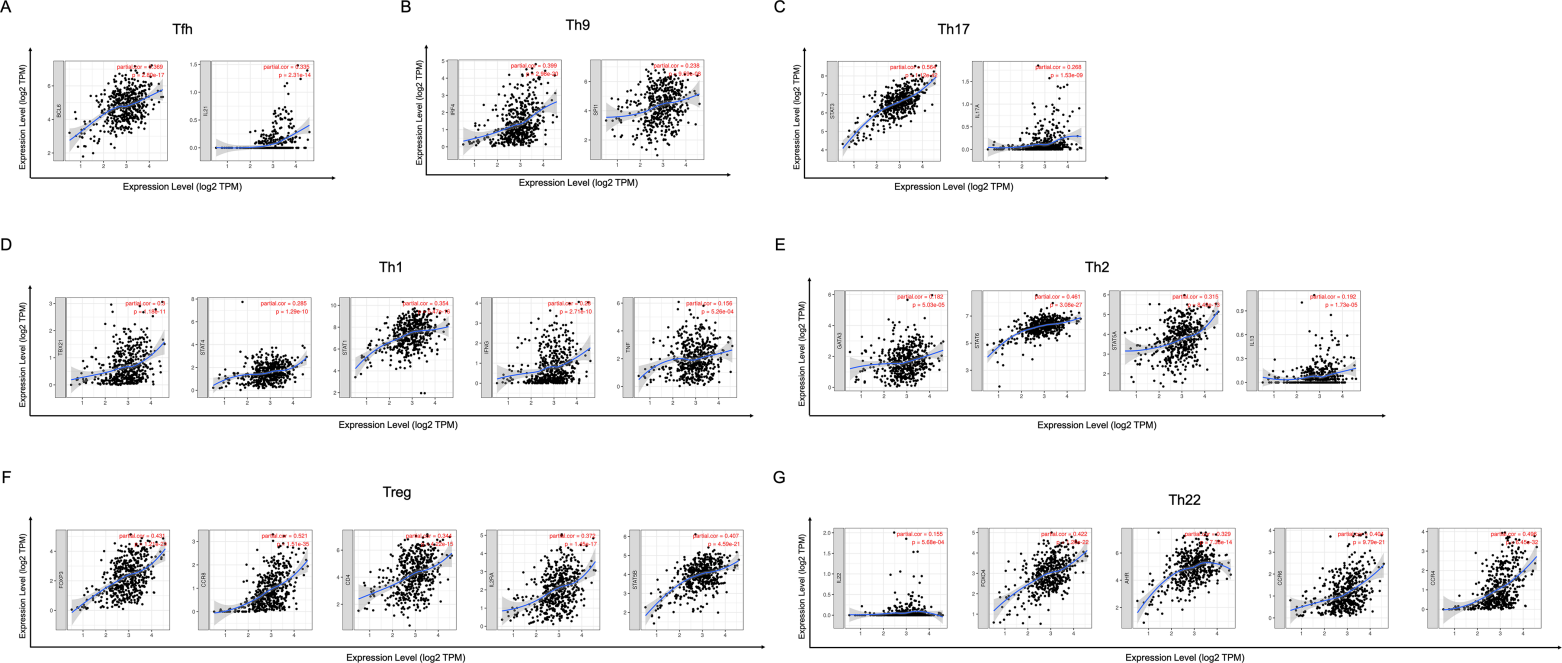
Scatterplots of correlations between YTHDC2 expression and gene markers of CD4+ T cells subsets. (A) Follicular helper T (Tfh) cells (B) T-helper 9 (Th9) cells (C) T-helper 17 (Th17) cells (D) T-helper 1 (Th1) cells (E) T-helper 2 (Th2) cells (F) Tregs (G) T-helper 22 (Th22) cells.

In order to study the relationship between YTHDC2 and different immune infiltrated cells, We used TIMER and GEPIA database simultaneously to study the correlation between YTHDC2 expression level in normal tissues and HNSCC tissues and immune marker sets in different immune cells. We found that the expression level of YTHDC2 was correlated with the immunomarker sets of most of the different T cells in HNSCC after the purity correlation adjustment. Notably, we found that the expression level of most CD4+ T cell subpopulation marker sets was closely related to the expression of YTHDC2 in HNSCC (Table 2). Specifically, the results showed STAT1 of T-helper 1 (Th1) cells, STAT6 of T-helper 2 (Th2) cells, BCL6 of follicular helper T (Tfh) cells, STAT3 of T-helper 17 (Th17) cells, FOXP3, CCR8, CD25 (IL-2R *α*) and STAT5B of Tregs, IRF4 of T-helper 9 (Th9) cells, FOXO4, CD196 (CCR6) and CD194 (CCR4) of T-helper 22 (Th22) cells are significantly correlated with YTHDC2 expression in HNSCC (Correlation > 0.35, *P* < 0.0001; Fig. 6). To further verify the above results, we analyzed the correlation between YTHDC2 expression and the above markers of CD4+ T cell subsets in the GEPIA database. The results are similar to the above TIMER results (Table 3). Therefore, these results confirm the specific correlation between YTHDC2 and HNSCC immune infiltrated cells.

**Table 1:** Correlation of YTHDC2 mRNA expression and overall survival in HNSCC with different clinicopathological factors by Kaplan–Meier plotter. Values styled in bold style indicate *P* < 0.05.

**Clinicopathologicalcharacteristics**	**Overall survival**		
	***N***	**Hazard ratio**	***P-*value**
**GENDER**			
Female	133	1.39 (0.85–2.29)	0.1908
Male	366	0.46 (0.31–0.71)	**0.00023**
**STAGE**			
1	25	0.19 (0.02–1.78)	0.1014
2	69	1.68 (0.57–4.94)	0.3403
3	78	0.63 (0.3–1.32)	0.2155
4	259	0.76 (0.54–1.08)	0.1266
**GRADE**			
1	61	2.52 (1.03–6.17)	**0.0371**
2	298	0.61 (0.4–0.92)	**0.0172**
3	119	0.58 (0.3–1.13)	0.1066
**MUTATION BURDEN**			
High	251	0.66 (0.44–0.99)	0.1091
Low	243	0.58 (0.38–0.88)	**0.0101**
**BASOPHILS**			
Enriched	243	0.64 (0.42–0.96)	**0.0287**
Decreased	254	0.65 (0.43–0.99)	**0.0452**
**B-CELLS**			
Enriched	238	0.37 (0.21–0.65)	**0.00035**
Decreased	259	1.31 (0.8–1.61)	0.4894
**CD4+ MEMORY CELLS**			
Enriched	316	0.55 (0.35–0.85)	**0.006**
Decreased	181	0.54 (0.33–0.89)	**0.0146**
**CD8+ T-CELLS**			
Enriched	305	0.7 (0.49–1)	**0.0489**
Decreased	192	1.32 (0.86–2.01)	0.1973
**EOSINOPHILS**			
Enriched	215	0.64 (0.42–0.96)	**0.0315**
Decreased	282	0.62 (0.4–0.95)	**0.0259**
**MACROPHAGES**			
Enriched	205	0.6 (0.38–0.96)	**0.0302**
Decreased	292	0.66 (0.46–0.95)	**0.0234**
**MESENCHYMALSTEM CELLS**			
Enriched	313	0.73 (0.52–1.03)	0.0723
Decreased	184	0.52 (0.33–0.8)	**0.0029**
**NATURAL KILLER T-CELLS**			
Enriched	129	0.66 (0.36–1.22)	0.1788
Decreased	368	0.69 (0.5–0.96)	**0.0258**
**REGULATORY T-CELLS**			
Enriched	322	0.68 (0.49–0.96)	**0.025**
Decreased	175	0.63 (0.38–1.06)	0.0775
**TH1 CELLS**			
Enriched	381	0.57 (0.39–0.82)	**0.002**
Decreased	116	1.31 (0.73–2.38)	0.3635
**TH2 CELLS**			
Enriched	473	0.65 (0.47–0.89)	**0.0064**
Decreased	24	0.23(0.04–1.48)	0.0967

### The prognostic signature built by the immune infiltration and the expression of YTHDC2

**Figure 7:**
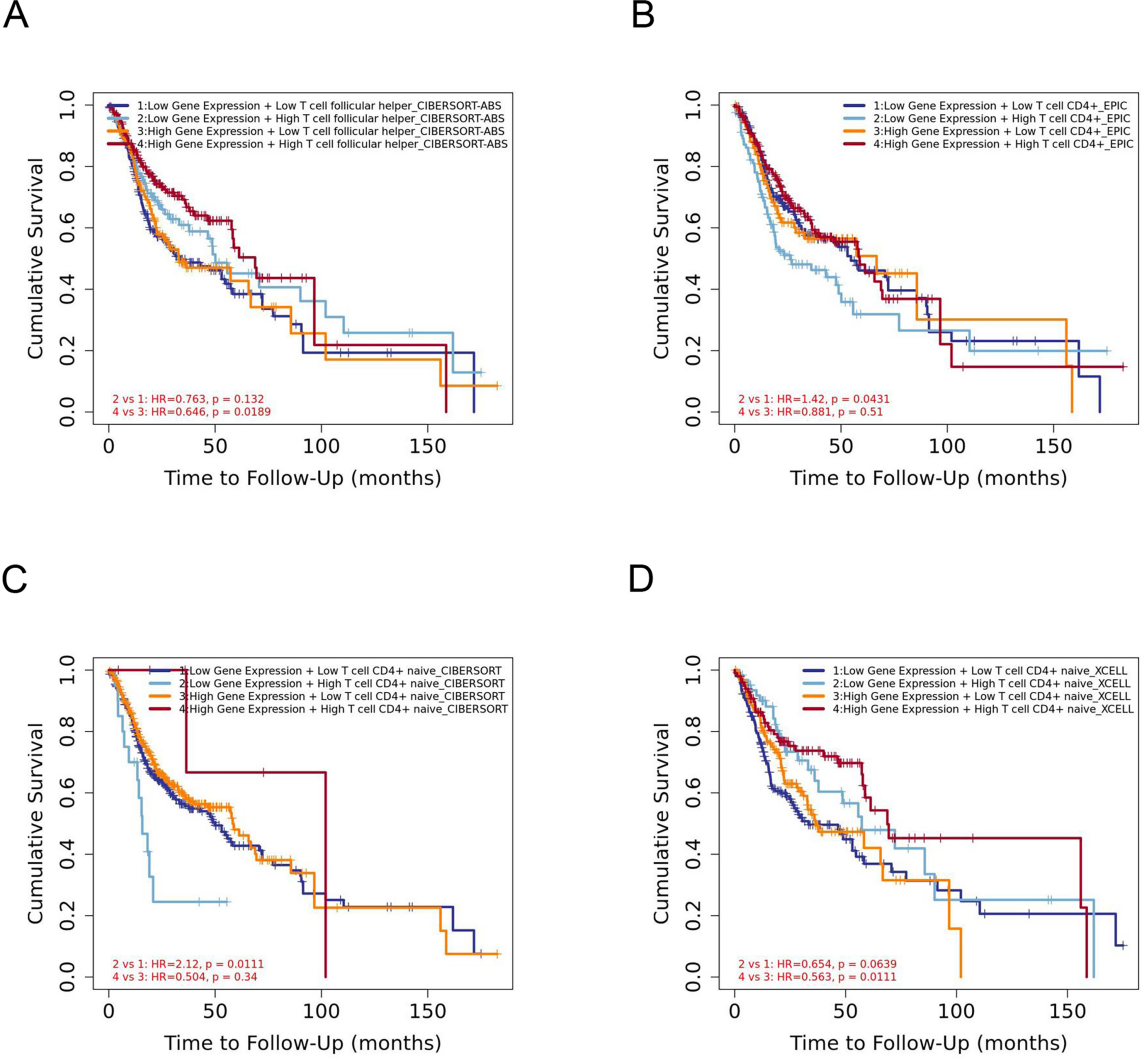
The prognostic signature built by the immune infiltration of CD4+ T cells subsets and the expression of YTHDC2. (A) The prognostic signature built by the immune infiltration of Tfh cells and the expression of YTHDC2 profiled by CIBERSORT-ABS algorithm. (B) The prognostic signature built by the immune infiltration of CD4+ T cells and the expression of YTHDC2 profiled by EPIC algorithm. (C) The prognostic signature built by the immune infiltration of CD4+ native T cells and the expression of YTHDC2 profiled by CIBERSORT algorithm. (D) The prognostic signature built by the immune infiltration of CD4+ native T cells and the expression of YTHDC2 profiled by xCell algorithm.

The infiltration level of CD4+ T cell subsets was divided into low and high levels and The covariate was selected as YTHDC2 expression. The hazard ratio and *P* value for Cox model were shown on the Kaplan–Meier survival curve plot. The results of Figs. 7A and 7D showed that high expression of YTHDC2 with high immune infiltration of Tfh cells or CD4+ native T cells have better prognosis than low expression of YTHDC2 with low immune infiltration of Tfh cells or CD4+ native T cells. However, the results of Figs. 7B and 7C showed that low YTHDC2 expression with low immune infiltration of CD4+ T cells or CD4+ native T cells have better prognosis than high expression of YTHDC2 with high immune infiltration of CD4+ T cells or CD4+ native T cells. However, it is not difficult to see that YTHDC2 with high expression level has a better prognosis than YTHDC2 with low expression level. Secondly, different CD4+ T cell subsets may have different effects on the prognosis of HNSCC.

**Table 2:** Correlation analysis between YTHDC2 and related genes and markers of immune cells in TIMER.

**Description**	**Gene markers**	**HNSCC**			
		**None**		**Purity**	
		**Cor**	***P***	**Cor**	***P***
CD8+ T cell	CD8A	0.349	***	0.352	***
	CD8B	0.262	***	0.253	***
T cell (general)	CD3D	0.236	***	0.228	***
	CD3E	0.334	***	0.337	***
	CD2	0.323	***	0.319	***
B cell	CD19	0.285	***	0.287	***
	CD79A	0.309	***	0.314	***
Monocyte	CD86	0.244	***	0.244	***
	CSF1R	0.299	***	0.301	***
TAM	CCL2	0.271	***	0.254	***
	CD68	0.090	0.04	0.086	0.058
	IL10	0.265	***	0.266	***
M1 Macrophage	NOS2	0.337	***	0.331	***
	IRF5	0.258	***	0.263	***
	PTGS2	0.130	*	0.129	*
M2 Macrophage	CD163	0.276	***	0.284	***
	VSIG4	0.196	***	0.196	***
	MS4A4A	0.215	***	0.213	***
Neutrophils	CEACAM8	0.046	0.293	0.045	0.321
	ITGAM	0.319	***	0.317	***
	CCR7	0.320	***	0.314	***
Natural killer cell	KIR2DL1	0.145	**	0.161	**
	KIR2DL3	0.247	***	0.243	***
	KIR2DL4	0.275	***	0.298	***
	KIR3DL1	0.275	***	0.289	***
	KIR3DL2	0.277	***	0.276	***
	KIR3DL3	0.134	*	0.120	*
	KIR2DS4	0.132	*	0.135	*
Dendritic cell	HLA-DPB1	0.238	***	0.236	***
	HLA-DQB1	0.222	***	0.207	***
	HLA-DRA	0.322	***	0.324	***
	HLA-DPA1	0.342	***	0.340	***
	CD1C	0.254	***	0.242	***
	NRP1	0.300	***	0.305	***
	ITGAX	0.293	***	0.308	***
Th1	TBX21	0.300	***	0.300	***
	STAT4	0.290	***	0.285	***
	STAT1	0.350	***	0.354	***
	IFN-*γ* (IFNG)	0.276	***	0.280	***
	TNF-*α* (TNF)	0.147	**	0.156	**
Th2	GATA3	0.190	***	0.182	***
	STAT6	0.460	***	0.461	***
	STAT5A	0.320	***	0.315	***
	IL13	0.199	***	0.192	***
Tfh	BCL6	0.363	***	0.369	***
	IL21	0.352	***	0.335	***
Th17	STAT3	0.573	***	0.564	***
	IL17A	0.277	***	0.268	***
Treg	FOXP3	0.422	***	0.431	***
	CCR8	0.519	***	0.521	***
	CD4	0.344	***	0.344	***
	CD25 (IL-2R*α*)	0.360	***	0.372	***
	STAT5B	0.408	***	0.407	***
Th9	IRF4	0.392	***	0.399	***
	PU.1 (SPL1)	0.234	***	0.238	***
Th22	IL22	0.167	**	0.155	**
	FOXO4	0.425	***	0.422	***
	AHR	0.329	***	0.329	***
	CD196 (CCR6)	0.401	***	0.404	***
	CD194 (CCR4)	0.498	***	0.496	***

**Notes.**

* *P* < 0.05.

** *P* < 0.01.

*** *P* < 0.001.

### Gene alteration of YTHDC2 in HNSCC from cBioPortal

**Figure 8:**
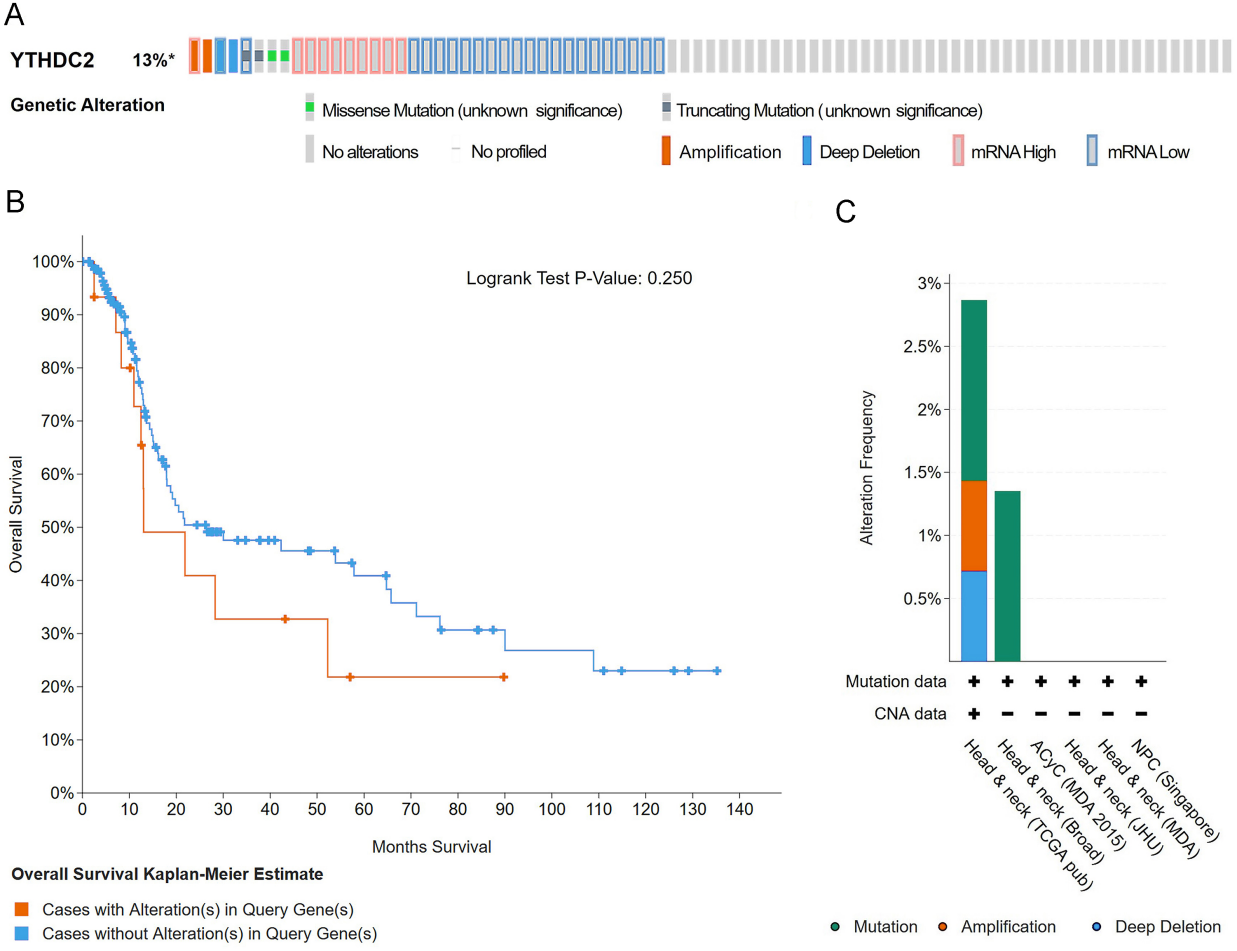
Gene alteration of YTHDC2 in head and neck squamous cell carcinoma (HNSCC). (A) The OncoPrint schematic shows the gene alteration of YTHDC2 in HNSCC. (B) The Kaplan–Meier survival curve shows no significant correlation between gene alteration of and the overall survival of patients in HNSCC. (C) Gene alteration frequency of YTHDC2 in HNSCC from different tissues.

The oncoprint schematic diagram showed gene alteration of YTHDC2 occurred in 37 of all the 279 sequenced cases (13%), including 3 cases of mutation, 1 cases of amplification, 1 case of deep deletion, 9 cases of mRNA up-regulation and 20 cases of mRNA down-regulation (Fig. 8A). However, the overall survival curve showed that there was no significant correlation between alteration of YTHDC2 and the overall survival of HNSCC patients (Fig. 8B). The statistical results of the frequency of gene alteration showed that YTHDC2 had a certain possibility of mutation (Fig. 8C).

## Discussion

Modification of m^6^A is a complex regulatory network and participates in various stages of the RNA life cycle, from RNA processing, nuclear output, translation regulation to RNA degradation, indicating that m^6^A has multiple functions related to RNA metabolism. Recent studies have shown that m^6^A methylation is closely related to the development and progression of tumors (Cui et al., 2017; Vu et al., 2017; Weng et al., 2018; Zhang et al., 2016). The evolutionarily conserved YTH domain contained in the YTH protein family selectively recognizes m6A. YTHDC2 is located on human chromosome 5 with a length of 81kb. The relative molecular weight of YTHDC2 protein is 160kD, containing 1430 amino acid sequences. Like other proteins in the YTH family, YTHDC2 contains a YTH domain at the C end, which is formed by six-stranded *β*-sheet and three *α*-helices with positively charged surface hydrophobic pockets, mediating binding to m6A (Theler et al., 2014; Xu et al., 2015). In addition, YTHDC2 differs from other YTH family proteins in that it has its own unique domain. Its RNA binding domain characterized R3H domain is involved in the binding of YTHDC2 to intracellular RNA, and plays an auxiliary role in the YTH domain. The Helicase domain of YTHDC2 has ATP-dependent RNA Helicase function, in which the ankyrin repeat (ANK) mediates the interaction with 5′-3′  exonuclease XRN1, suggesting that YTHDC2 may play a role in regulating mRNA stability (Kretschmer et al., 2018). However, the function of DUF1065 domain of YTHDC2 is not yet clear and needs further study. As the final member of the YTH protein family, the biological function of YTHDC2 is still not fully understood. Kretschmer et al. (2018) showed that YTHDC2 is mainly enriched in the perinuclear region and can bind to ribosomes and act on small ribosomal subunits to improve translation efficiency. Wojtas et al. (2017) found that YTHDC2 has the activity of 3′→5′  RNA helicase, which can regulate the transcription level of m6A-modified germline, so as to maintain the gene expression program of meiosis process. Other studies have shown that YTHDC2 may be associated with autism (Liu et al., 2016), and may also be a potential susceptibility gene for pancreatic cancer (Fanale et al., 2014). It also plays an important role in the occurrence and development of liver cancer (Tanabe et al., 2014), but the relationship between YTHDC2 and HNSCC has been rarely studied.

**Table 3:** Correlation analysis between YTHDC2 and related genes and markers of CD4+ T cells subsets in GEPIA.

**Description**	**Gene markers**	**HNSCC**			
		**Tumor**		**Normal**	
		**R**	***P***	**R**	***P***
Th1	TBX21	0.28	***	0.14	0.37
	STAT4	0.27	***	0.25	0.11
	STAT1	0.36	***	0.48	**
	IFN- *γ* (IFNG)	0.25	***	0.11	0.47
	TNF- *α* (TNF)	0.16	**	0.29	0.055
Th2	GATA3	0.2	***	−0.11	0.47
	STAT6	0.5	***	0.87	***
	STAT5A	0.33	***	0.085	0.58
	IL13	0.21	***	−0.044	0.78
Tfh	BCL6	0.41	***	0.4	*
	IL21	0.35	***	0.24	0.12
Th17	STAT3	0.59	***	0.64	***
	IL17A	0.26	***	0.41	*
Treg	FOXP3	0.35	***	0.3	0.045
	CCR8	0.49	***	0.29	0.052
	CD4	0.33	***	0.18	0.25
	CD25 (IL2RA)	0.32	***	0.16	0.29
	STAT5B	0.42	***	0.091	0.55
Th9	IRF4	0.37	***	0.051	0.74
	PU.1 (SPI1)	0.2	***	0.02	0.9
Th22	IL22	0.2	**	0.47	*
	FOXO4	0.45	***	0.21	0.17
	AHR	0.35	***	0.65	***
	CD196 (CCR6)	0.34	***	0.33	0.027
	CD194 (CCR4)	0.45	***	0.29	0.054

**Notes.**

* *P* < 0.05.

** *P* < 0.01.

*** *P* < 0.001.

Head and neck tumors are the sixth most common type of malignancy in the world, with HNSCC accounting for 90%. There are more than 550,000 new cases and 380,000 deaths worldwide each year. According to the TNM stage of the tumor, the current treatment methods of HNSCC mainly include surgical treatment, radiotherapy and chemotherapy, but the treatment effect is still not satisfactory.

In this study, to investigate the role of YTHDC2 in HNSCC, gene co-expression network was established to verify whether YTHDC2 was related to the prognosis of HNSCC. We found that YTHDC2 appeared in the blue module related to survival time and survival state. Later, we verified it again in the public database, and we found that there was a close correlation between YTHDC2 and the prognosis of HNSCC. Through this study, we found that YTHDC2 is a tumor suppressor gene with high expression in normal tissues and low expression in tumor tissues through IHC analysis. In addition, the expression of YTHDC2 was significantly correlated with immune infiltration level and had a high mutation frequency. Our results demonstrated that there was a positive relationships between YTHDC2 expression level and infiltration level of CD4+ T cell subsets in HNSCC. However there are few articles reported on the correlation between YTHDC2 expression level and infiltration level of CD4+ T cell subsets in HNSCC. At present, studies have shown that m6A modification plays an important role in the diversity and complexity of tumor microenvironment (Zhang et al., 2020). He et al. (2020) revealed that high RNA methylation modification group was associated with significantly higher numbers of tumor-infiltrating CD8+ T cells, Th cells and activated NK cells, but lower expressions of PD-L1, PD-L2, TIM3, and CCR4 than low RNA methylation modification group in breast cancer patients. Li et al. (2020a) found low WTAP expression was associated with a high T cell-related immune response, and lymphocyte infiltration in patients with low WTAP expression was well correlated with the prognosis of gastric cancer. Besides, current study has found *α*-ketoglutarate-dependent dioxygenase alkB homolog 5 (ALKBH5) can modulate the composition of tumor-infiltrating Tregs. The mutation and expression status of ALKBH5 correlate with their response to immunotherapy in melanoma patients (Li et al., 2020b). In this study, we found STAT1 of Th1 cells, STAT6 of Th2 cells, BCL6 of Tfh cells, STAT3 of Th17 cells, FOXP3, CCR8, CD25 (IL-2R) and STAT5B of Tregs, IRF4 of Th9 cells, FOXO4,CD196 (CCR6) and CD194 (CCR4) of Th22 cells are significantly correlated with YTHDC2 expression in HNSCC. Further experiments are needed to prove the correlation between YTHDC2 and CD4+ T cell subsets and the specific mechanism.

## Conclusions

In conclusion, we found that m^6^A methylation regulator YTHDC2 may become a potential marker for molecular diagnosis and prognosis of HNSCC through this study, and will also provide a new target for the development of clinical molecular targeted therapy drugs. In addition, the expression of YTHDC2 was positively correlated with infiltrating levels of CD4+ T cells subsets in HNSCC. However, the specific mechanism of YTHDC2 acting on HNSCC needs further exploration.

##  Supplemental Information

10.7717/peerj.10385/supp-1 Supplemental Information 1 Clinical traits

10.7717/peerj.10385/supp-2 Supplemental Information 2 Input data

10.7717/peerj.10385/supp-3 Supplemental Information 3 Raw data exported from the public databases applied for data analyses and preparation for Figs. 4, 5 and 8 and Table 2.

10.7717/peerj.10385/supp-4 Supplemental Information 4 Raw data exported from the public databases applied for data analyses and preparation for Fig. 6

10.7717/peerj.10385/supp-5 Supplemental Information 5 Raw data exported from the source code of weighted gene co-expression network analysis

## Abbreviations

ALKBH5: *α*-ketoglutarate-dependent dioxygenase alkB homolog 5
ANK: ankyrin repeat
DAB: diaminobenzidine
DCs: dendritic cells
EGA: European Genome-phenome Archive
GEO: Gene Expression Omnibus
GEPIA: Gene expression Profling Interactive Analysis
GS: gene significance
GTEx: Genotype-Tissue Expression
HNRNPC: heterogeneous nuclear ribonucleoprotein C
HNSCC: head and neck squamous cell carcinoma
HR: hazard ratio
IHC: Immunohistochemistry
m6A: N6-methyladenosine
METTL3: methyltransferase like 3
MM: membership module
NK: natural killer
PBS: phosphate buffer saline
PFS: progression-free survival
RFS: recurrence-free survival
TCGA: The Cancer Genome Atlas
Th1: T-helper 1
Th2: T-helper 2
Th9: T-helper 9
Th17: T-helper 17
Th22: T-helper 22
WATP: wilm’s tumor 1-associating protein
WGCNA: weighted gene co-expression network analysis
Tfh: follicular helper T
YTH: YT521-B homology
YTHDCl-2: YTH domain-containing 1-2
YTHDFl-3: YTH N6-methyladenosine RNA binding protein 1-3

## Additional Information and Declarations

### Data Availability

The following information was supplied regarding data availability: The data is available at NCBI GEO: GSE65858 and at the Head and Neck Squamous Cell Carcinoma (TCGA, Nature 2015). The original files of other datasets are available in the Supplemental Files.
