# Supplementary figures and images for: The m6A reader protein YTHDC2 is a potential biomarker and associated with immune infiltration in head and neck squamous cell carcinoma
Source: PeerJ. 2020 Nov 26;8:e10385. doi: 10.7717/peerj.10385 (PMC7700739; doi:10.7717/peerj.10385)

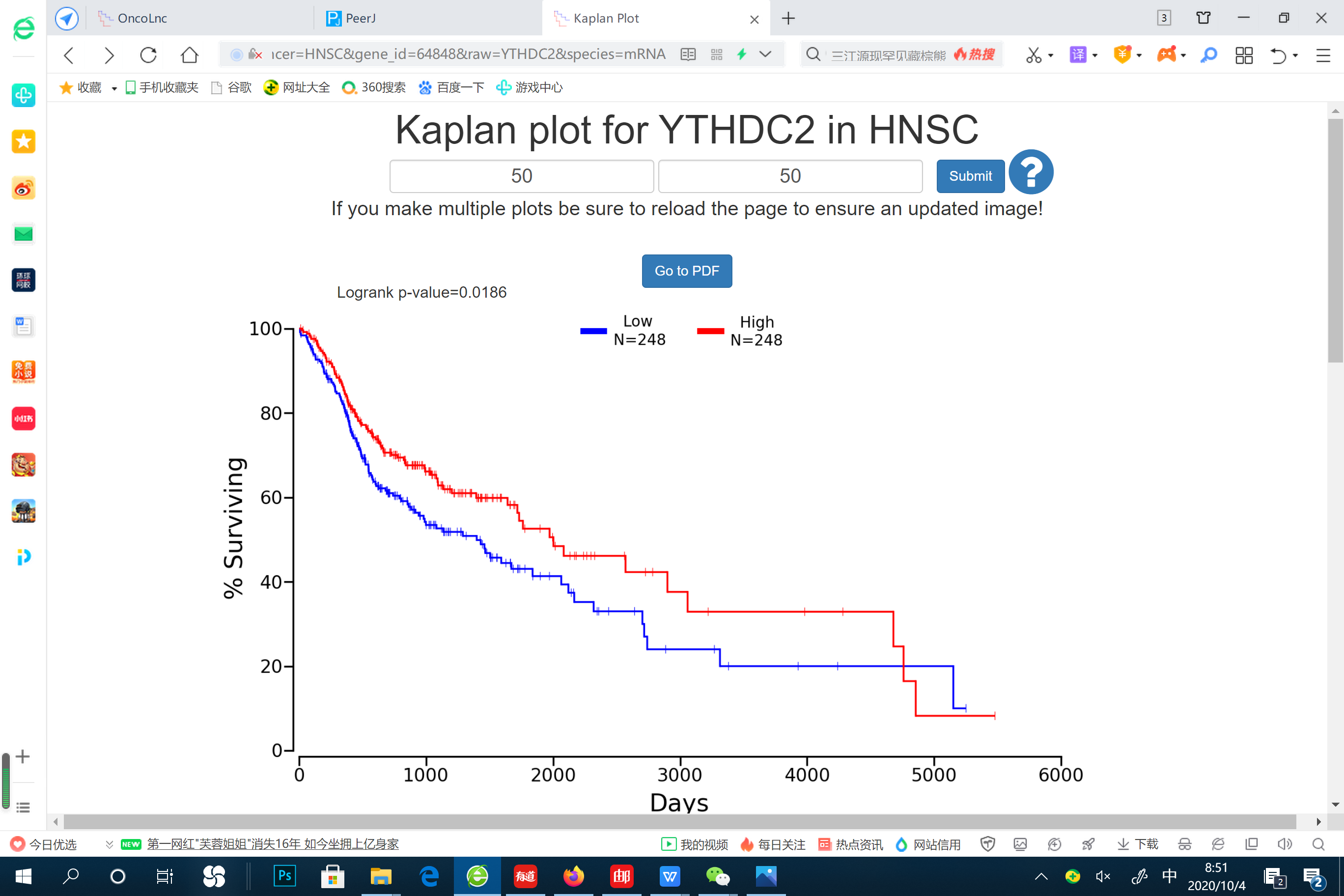

Supplement: Supplemental Information 3 [file peerj-08-10385-s003.zip › Raw data 3/dataset 1/3.png]

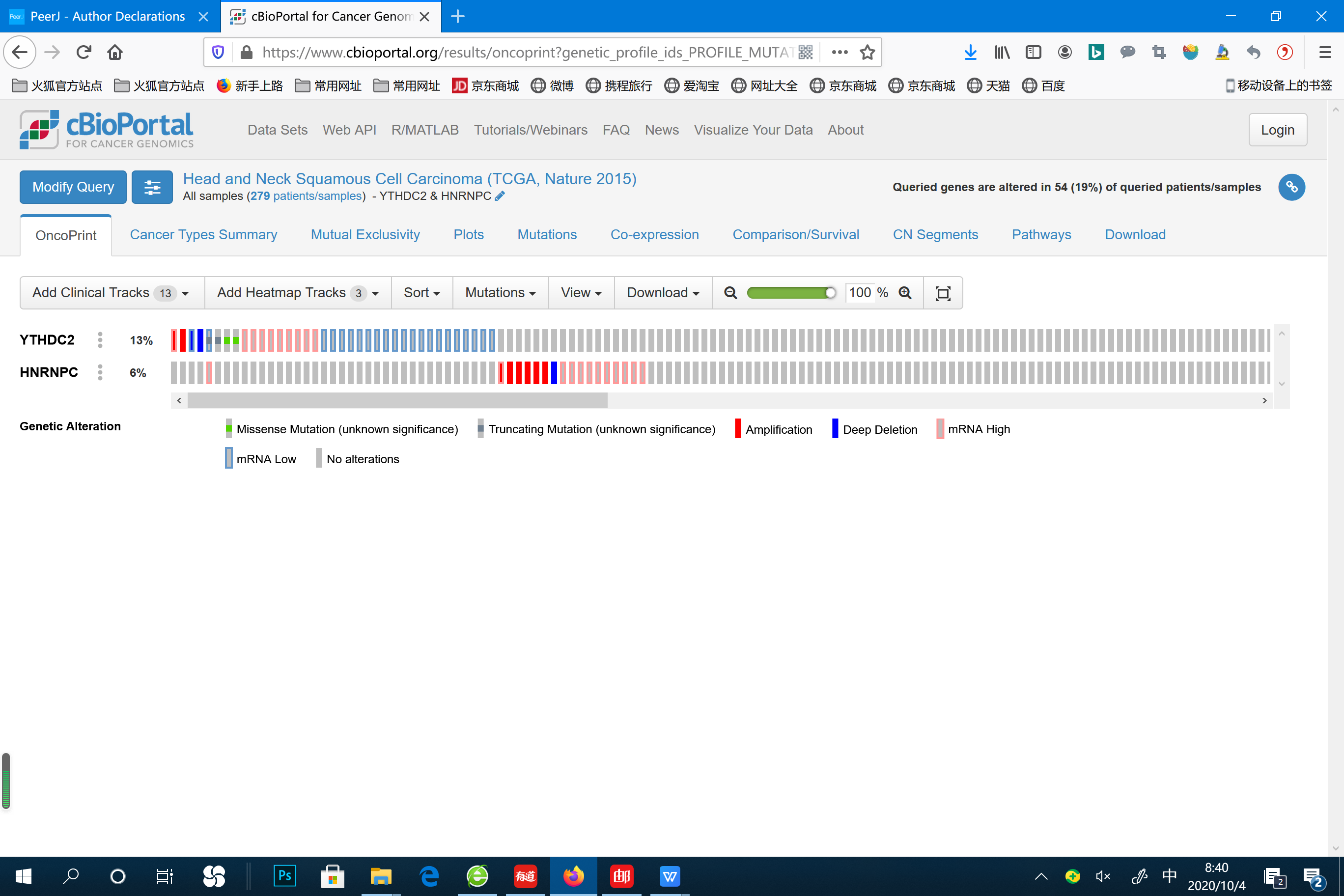

Supplement: Supplemental Information 3 [file peerj-08-10385-s003.zip › Raw data 3/dataset 1/4.png]

# YTHDC2

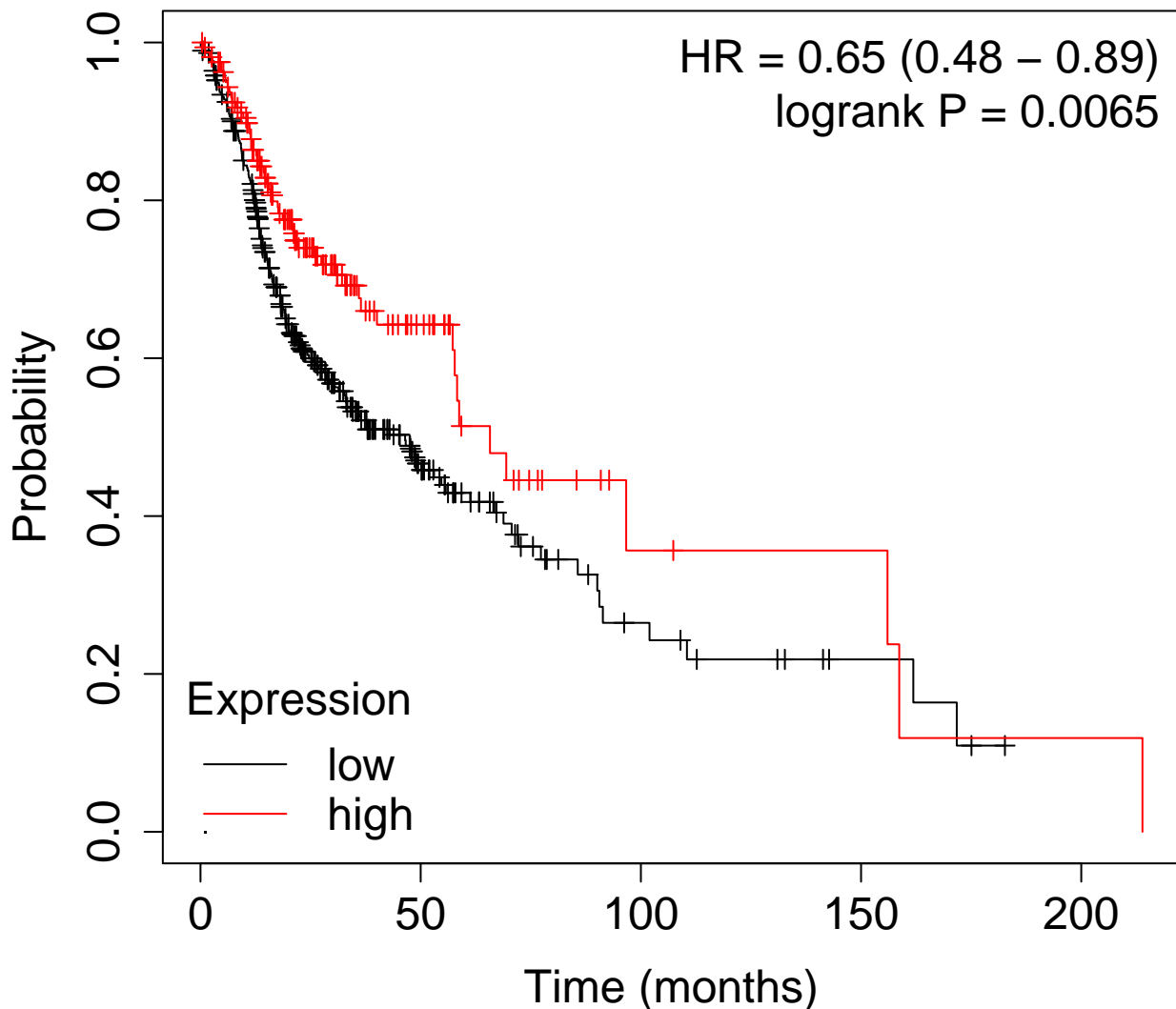

Number at risk

low  
high

336  
163

57  
29

12  
4

4  
3

0  
1

Supplement: Supplemental Information 3 [file peerj-08-10385-s003.zip › Raw data 3/dataset 1/5.pdf]

# YTHDC2

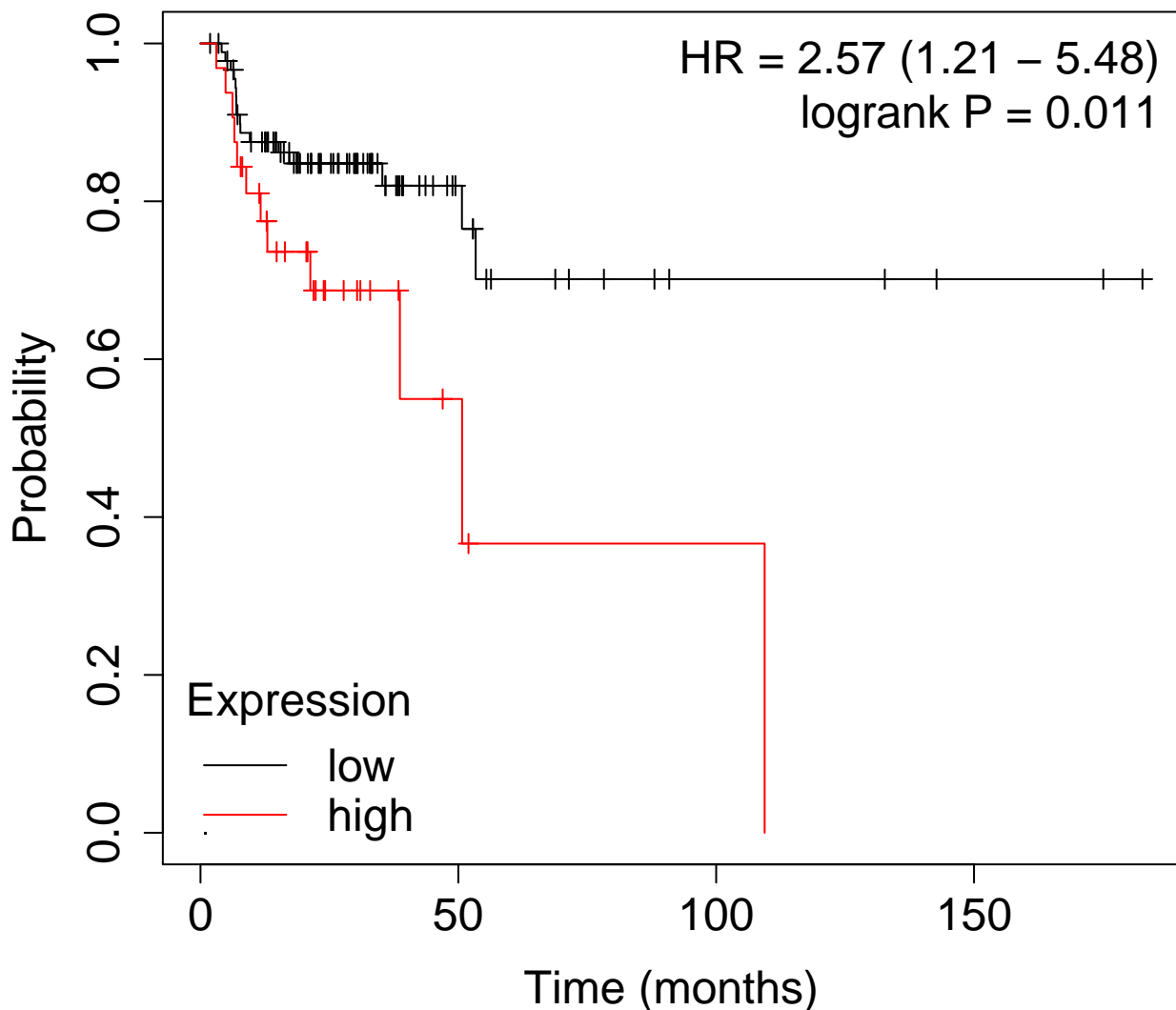

Number at risk

low  
high

92  
32

15  
3

4  
1

2  
0

Supplement: Supplemental Information 3 [file peerj-08-10385-s003.zip › Raw data 3/dataset 1/6.pdf]

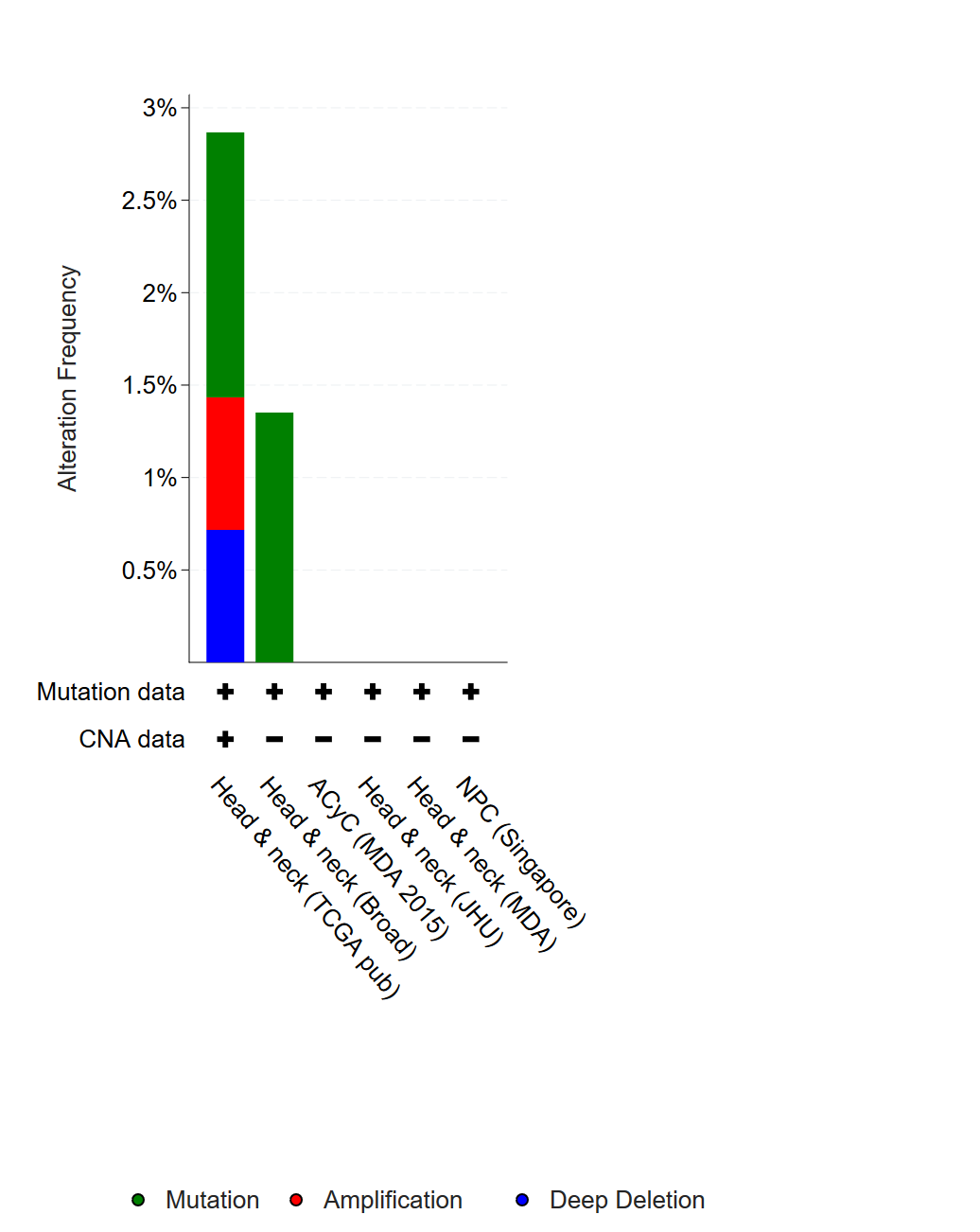

Supplement: Supplemental Information 3 [file peerj-08-10385-s003.zip › Raw data 3/dataset 1/7.png]

YTHDC2 Expression Level (log2 RSEM)

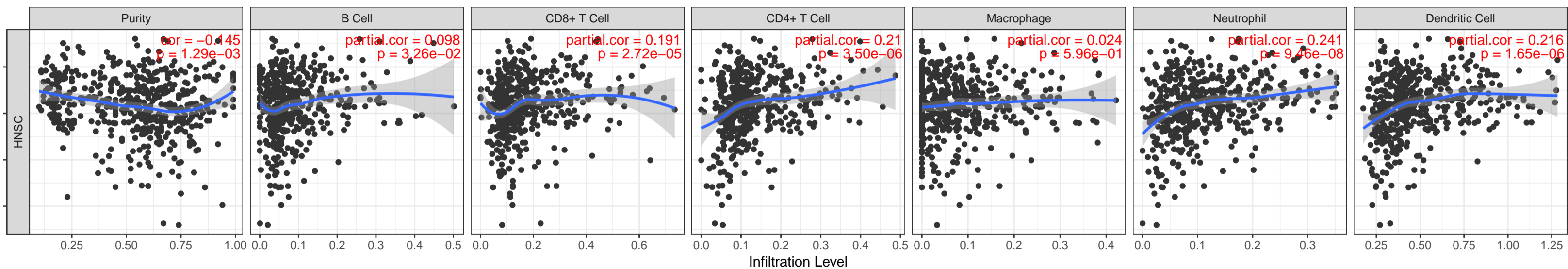

Supplement: Supplemental Information 3 [file peerj-08-10385-s003.zip › Raw data 3/dataset 1/8.pdf]

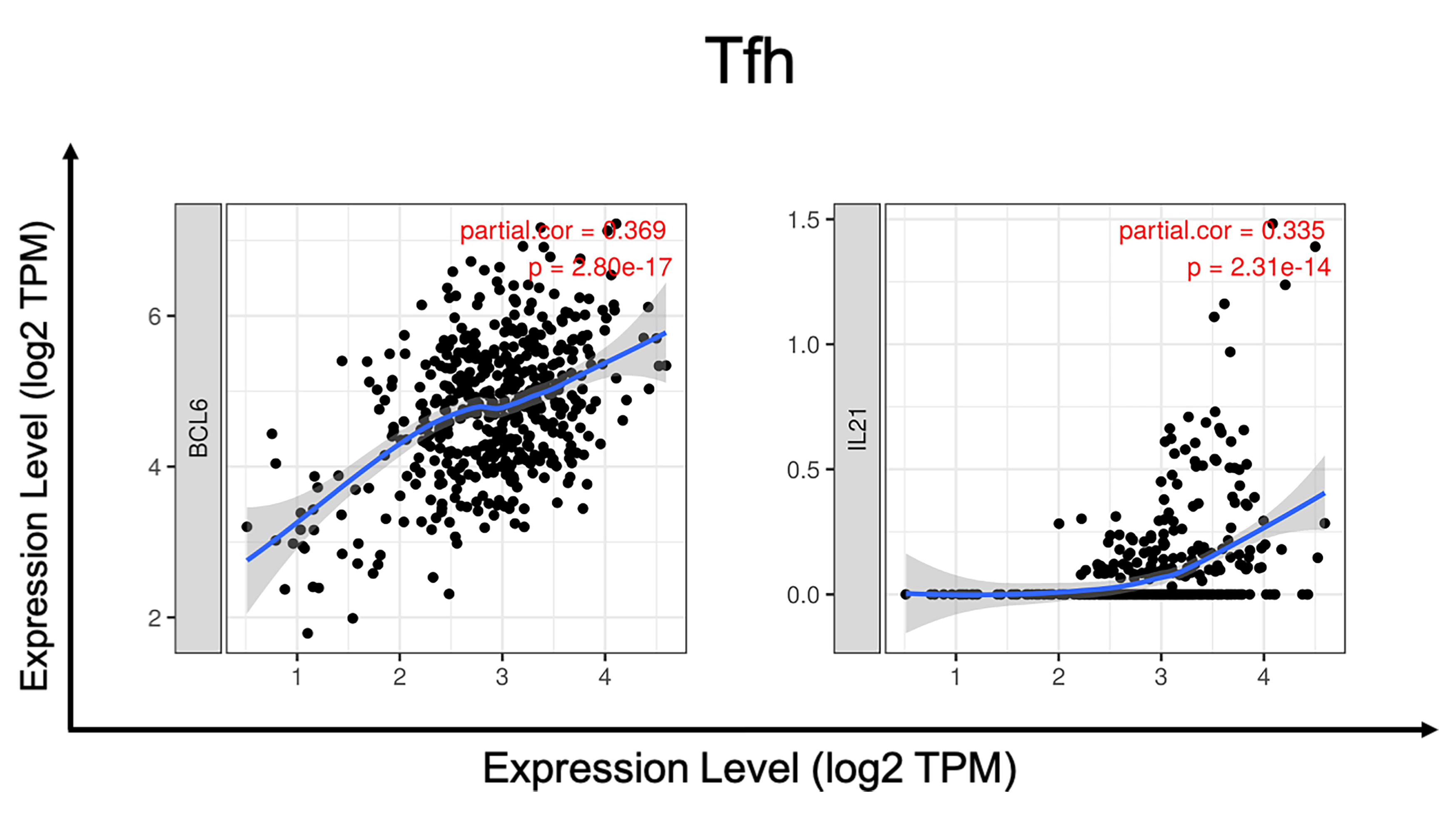

Supplement: Supplemental Information 4 [file peerj-08-10385-s004.zip › Raw data 4/dataset 2/Tfh.jpg]

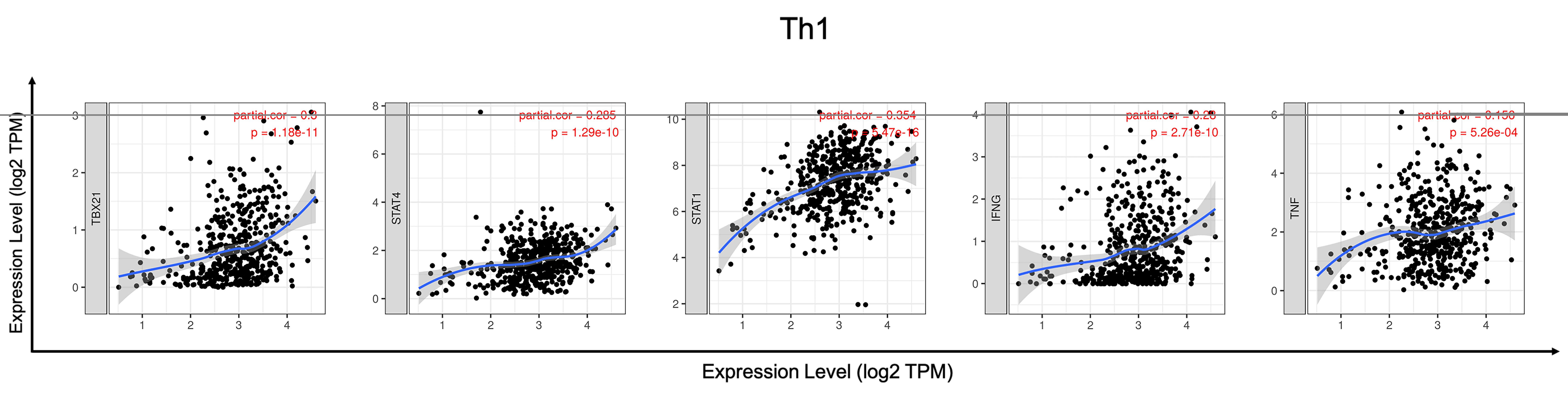

Supplement: Supplemental Information 4 [file peerj-08-10385-s004.zip › Raw data 4/dataset 2/Th1.jpg]

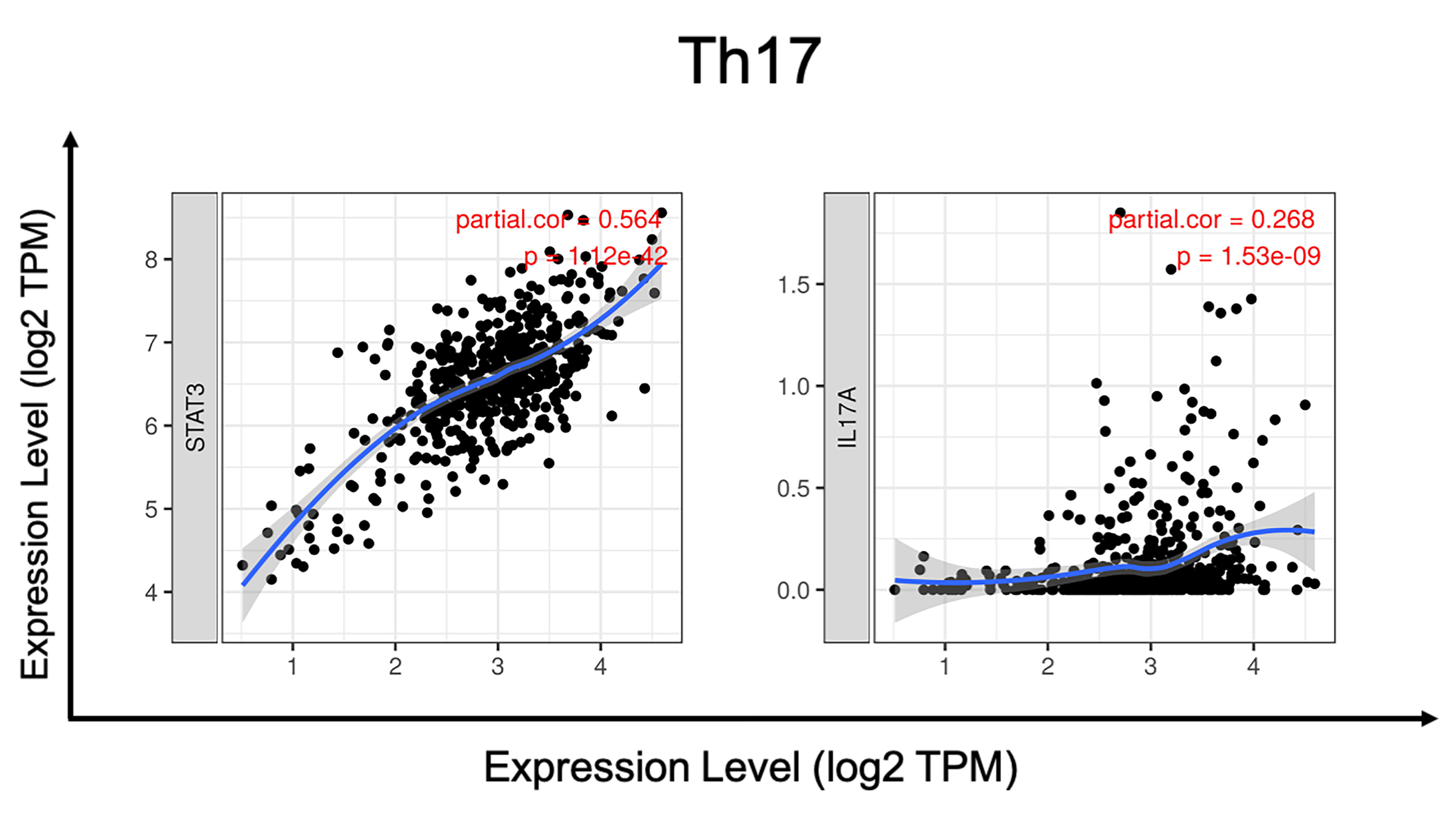

Supplement: Supplemental Information 4 [file peerj-08-10385-s004.zip › Raw data 4/dataset 2/Th17.jpg]

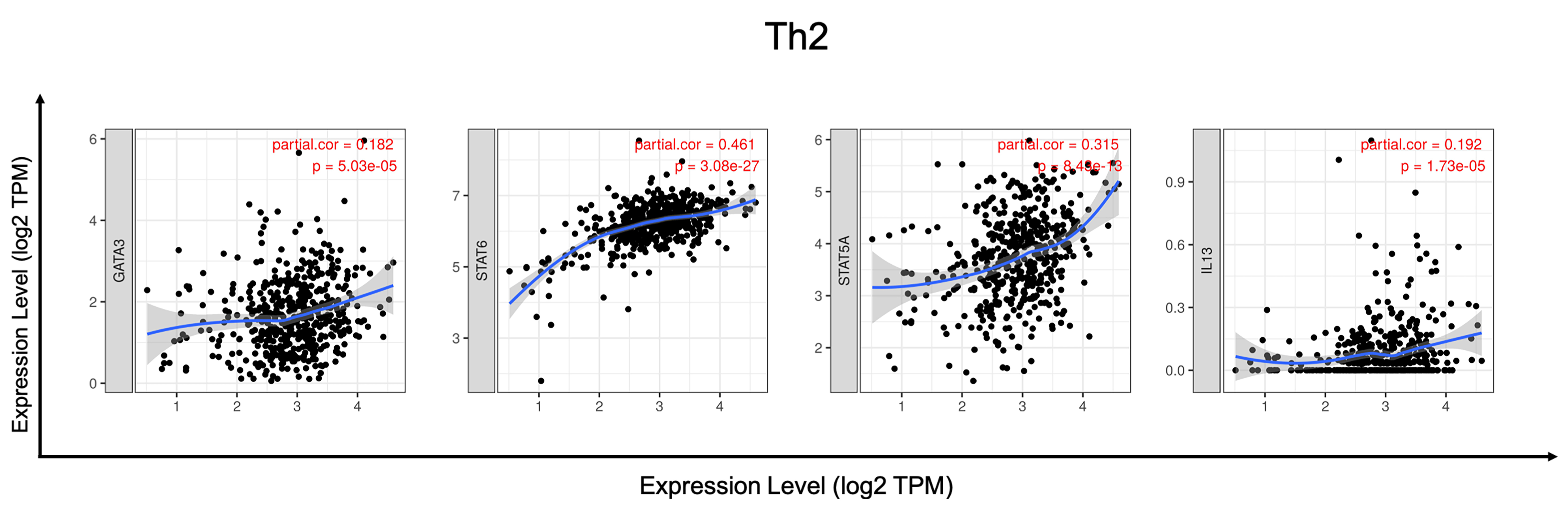

Supplement: Supplemental Information 4 [file peerj-08-10385-s004.zip › Raw data 4/dataset 2/Th2.jpg]

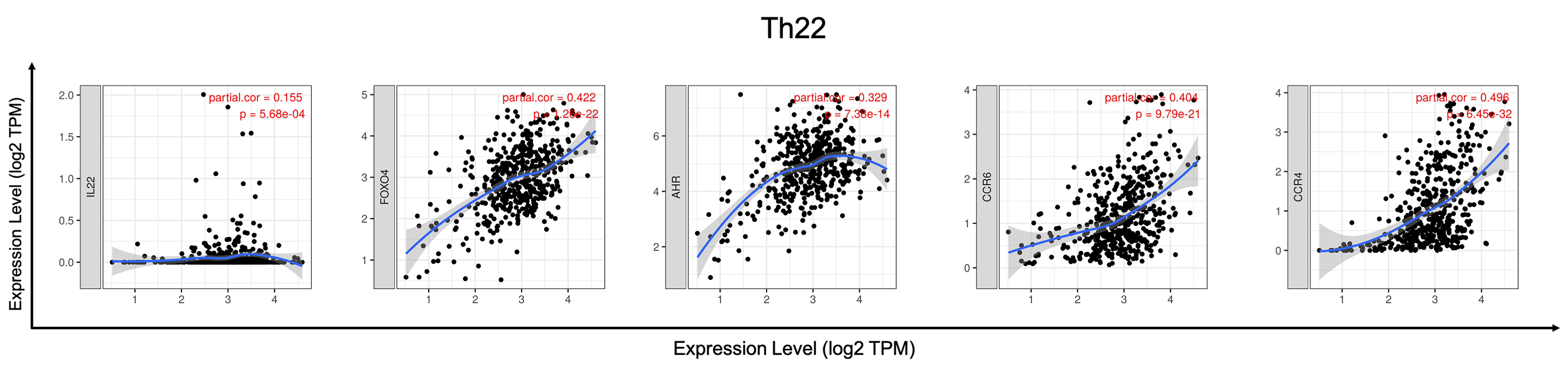

Supplement: Supplemental Information 4 [file peerj-08-10385-s004.zip › Raw data 4/dataset 2/Th22.jpg]

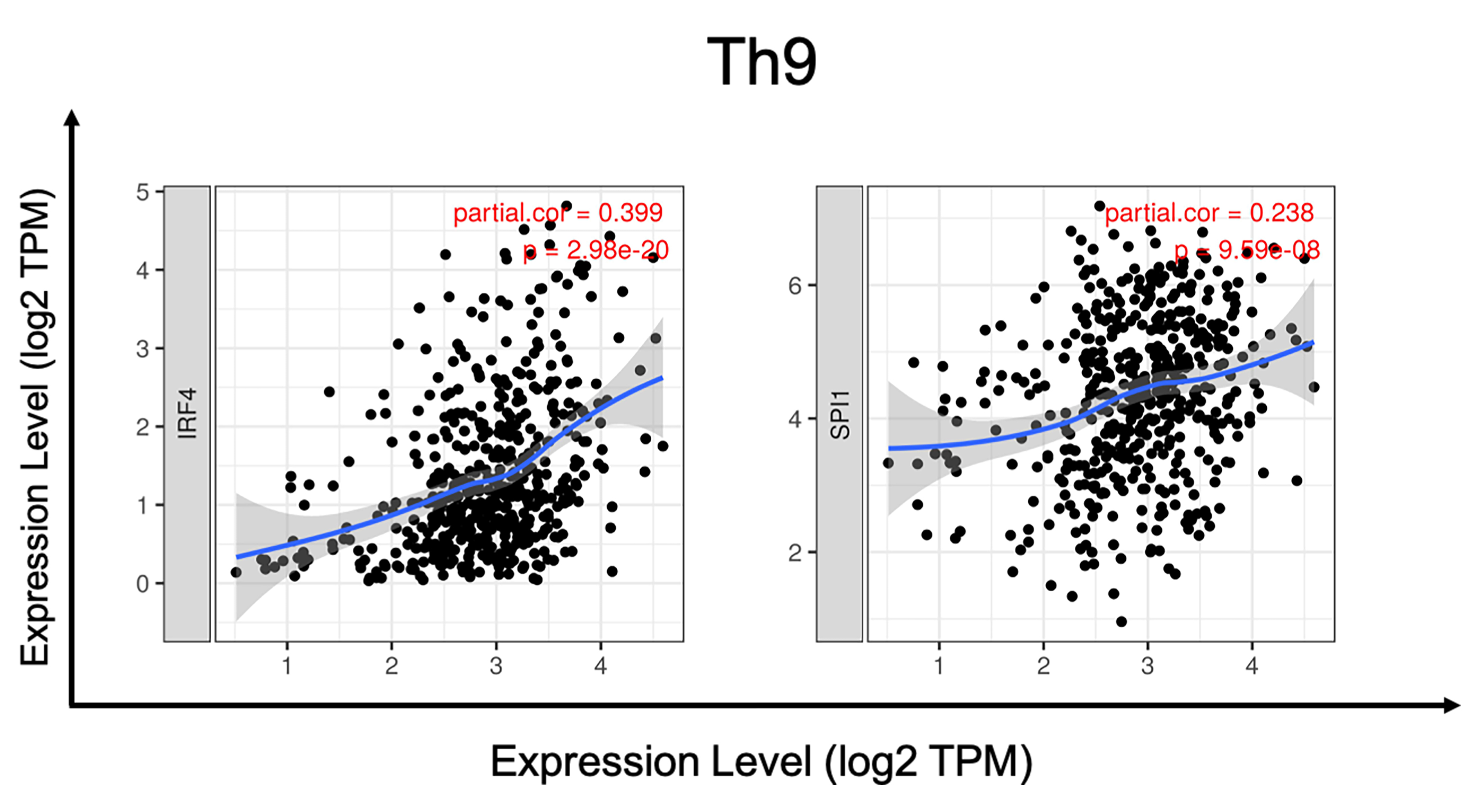

Supplement: Supplemental Information 4 [file peerj-08-10385-s004.zip › Raw data 4/dataset 2/Th9.jpg]

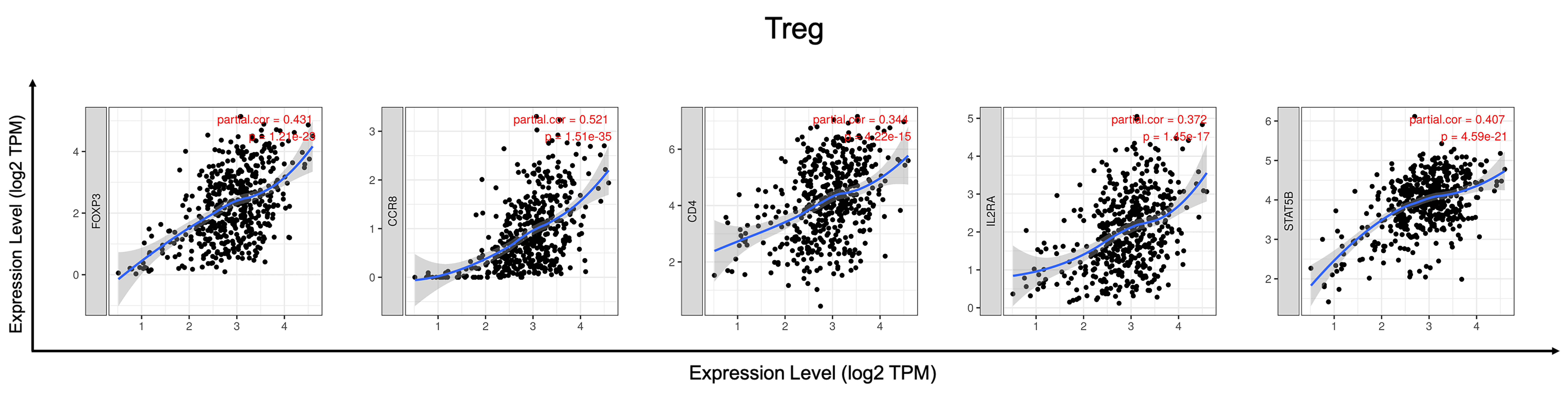

Supplement: Supplemental Information 4 [file peerj-08-10385-s004.zip › Raw data 4/dataset 2/Treg.jpg]
